# Assessment of Patient-Specific Human Leukocyte Antigen Genomic Loss at Relapse After Antithymocyte Globulin–Based T-Cell–Replete Haploidentical Hematopoietic Stem Cell Transplant

**DOI:** 10.1001/jamanetworkopen.2022.6114

**Published:** 2022-04-06

**Authors:** Hengwei Wu, Jimin Shi, Yi Luo, Jian Yu, Xiaoyu Lai, Lizhen Liu, Huarui Fu, Guifang Ouyang, Xiaojun Xu, Haowen Xiao, He Huang, Yanmin Zhao

**Affiliations:** 1 Bone Marrow Transplantation Center, The First Affiliated Hospital, Zhejiang University School of Medicine, Hangzhou, Zhejiang, People’s Republic of China; 2 Institute of Hematology, Zhejiang University, Hangzhou, Zhejiang, People’s Republic of China; 3 Zhejiang Province Engineering Laboratory for Stem Cell and Immunity Therapy, Zhejiang University, Hangzhou, Zhejiang, People’s Republic of China; 4 Zhejiang Laboratory for Systems and Precision Medicine, Zhejiang University Medical Center, Hangzhou, Zhejiang, People’s Republic of China; 5 Department of Hematology, Ningbo Hospital of Zhejiang University, Ningbo, China, Ningbo, Zhejiang, People’s Republic of China; 6 Department of Hematology-Oncology, The Children’s Hospital, Zhejiang University School of Medicine, Hangzhou, Zhejiang, People’s Republic of China; 7 Department of Hematology, The Sir Run Run Shaw Hospital, Zhejiang University School of Medicine, Hangzhou, Zhejiang, People’s Republic of China

## Abstract

**Importance:**

Patient-specific human leukocyte antigen (HLA) genomic loss (HLA loss) is one of the reputed mechanisms of leukemia immune escape and relapse after haploidentical hematopoietic stem cell transplant (HSCT). However, clinical characteristics and prognosis of this distinct relapse type in the setting of haploidentical HSCT based on antithymocyte globulin (ATG) T-cell–replete conditioning are still unknown, especially for patients with lymphoid leukemia.

**Objective:**

To identify the incidence of and patient characteristics associated with HLA loss at hematologic cancer relapse after ATG-based haploidentical HSCT and to assess overall survival after HLA loss at relapse.

**Design, Setting, and Participants:**

This retrospective and multicenter case series study used data from medical records to identify patients who experienced relapse of hematologic cancer after receipt of ATG-based haploidentical HSCT. The study included 788 consecutive patients aged 8 to 70 years with lymphoid or myeloid leukemia who received ATG T-cell–replete haploidentical HSCT at the Zhejiang Cooperative Group for Blood and Marrow Transplantation between May 1, 2012, and May 31, 2021.

**Exposures:**

Relapse after ATG-based haploidentical HSCT.

**Main Outcomes and Measures:**

Incidence, risk factors, and postrelapse overall survival among patients with HLA loss at hematologic cancer relapse after receipt of haploidentical HSCT. Logistic regression analysis was used to identify characteristics associated with the likelihood of HLA loss, and Kaplan-Meier and Cox regression analyses were performed to evaluate postrelapse survival.

**Results:**

A total of 788 patients who received haploidentical HSCT for hematologic cancer were identified, 180 of whom experienced relapse after HSCT. Of those, 106 evaluable patients (median age, 30.9 years [range, 8.3-64.6 years]; 54 female [50.9%] and 52 male [49.1%]) were screened for HLA loss, which was detected in 54 patients (50.9%). Patients with HLA loss experienced relapse later than those without HLA loss (lymphoid group: median, 323 days [range, 98-2056 days] vs 151 days [range, 57-2544 days]; *P* = .01; myeloid group: median, 321 days [range, 55-1574 days] vs 223 days [range, 68-546 days]; *P* = .03). Among patients with lymphoid leukemia, those with minimal residual disease positivity before hematologic relapse (odds ratio [OR], 28.47; 95% CI, 1.99-407.98; *P* = .01), those with chronic graft-vs-host disease (OR, 27.68; 95% CI, 1.40-546.88; *P* = .03), and those with more than 180 days between HSCT and relapse (OR, 6.91; 95% CI, 1.32-36.22; *P* = .02) were more likely to lose unshared HLA at relapse, whereas male patients (OR, 0.03; 95% CI, 0.003-0.32; *P* = .04) were more likely to preserve their HLA genome at relapse. Patients with myeloid leukemia had different factors associated with HLA loss, including underweight status (OR, 0.10; 95% CI, 0.02-0.60; *P* = .01) and acute graft-vs-host disease (OR, 4.84; 95% CI, 1.14-20.53; *P* = .03). The receipt of preemptive donor lymphocyte infusion among patients with minimal residual disease recurrence did not postpone hematologic cancer relapse in those with HLA loss (median, 322 days [range, 204-1030 days]) compared with no receipt of donor lymphocyte infusion (median, 340 days [range, 215 days to not available]; *P* > .99).

**Conclusions and Relevance:**

In this study, HLA loss at leukemia relapse occurred frequently after receipt of ATG-based haploidentical HSCT. The identification of risk factors associated with HLA loss would help to prompt screening, avoid potentially harmful infusions of donor T cells, and develop alternative therapeutic strategies.

## Key Points

**Question:**

What are the incidence, risk factors, and overall survival among patients with myeloid and lymphoid leukemia who experience human leukocyte antigen (HLA) loss at relapse after receipt of haploidentical hematopoietic stem cell transplant (HSCT)?

**Findings:**

In this case series study of 788 patients with myeloid and lymphoid leukemia who received HSCT, HLA loss occurred in 50.9% of patients experiencing hematologic cancer relapse. Patients with HLA loss at relapse had distinct characteristics, such as chronic or acute graft-vs-host disease, minimal residual disease before relapse, greater than 180 days between HSCT and relapse, or overweight status, compared with those without HLA loss at relapse.

**Meaning:**

This study found a high incidence of HLA loss at relapse after haploidentical HSCT, which may serve as an incentive to screen patients for factors associated with HLA loss and develop alternative therapeutic strategies.

## Teaser

This case series study examines incidence, risk factors, and overall survival among patients with lymphoid and myeloid leukemia who experienced human leukocyte antigen genomic loss at cancer relapse after receipt of haploidentical hematopoietic stem cell transplant.

## Introduction

Allogeneic hematopoietic stem cell transplant (HSCT) is a curative strategy for hematologic cancer because of the graft-vs-leukemia (GVL) effect derived from the donor immune system.^1^ With the development of prophylaxis strategies (posttransplant cyclophosphamide and antithymocyte globulin), haploidentical hematopoietic stem cell transplant is coming of age, providing patients who lack fully matched donors with opportunities for transplant while creating a more intense GVL effect. The GVL effect is based on the capability of immune cells to engage with tumor-specific antigens, minor histocompatibility antigens, and mismatched human leukocyte antigens (HLAs) on the leukemic cells.^2^ Although the interaction between 2 immune systems with incompatible HLAs poses the risk of graft-vs-host disease (GVHD), incompatible HLAs are also a dispensable source of the GVL effect in haploidentical HSCT.^3^ However, relapse has continued to surpass toxic effects on organs, infectious events, and GVHD, becoming the predominant cause of death after HSCT.^4^

The biological processes associated with posttransplant relapse have intrigued hematologists for decades. In particular, the downregulation or loss of unshared HLA molecules has been suggested as a rational reason for relapse because of the inability of T cells to meditate alloreactivity normally.^5,6,7^ Several studies^6,8^ observed altered HLA expression in a variety of solid tumors; this altered expression has long been thought to account for tumor progression due to impaired presentation of neoantigens. In contrast, HLA regions seldom change in hematologic neoplasms, especially at the initial stage; however, alterations progressively increase in patients who experience relapse after allogenic HSCT.^9,10,11^

After haploidentical HSCT, approximately one-third of patients with myeloid leukemia who experience relapse lose the patient-specific HLA genome in their leukemic cells.^12^ In addition, researchers have observed that loss of unshared HLA during relapse was infrequent in patients receiving transplants from well-matched and partially matched unrelated donors compared with haploidentical HLA donors.^13^ Thus, hematologists have speculated that loss of patient-specific HLA molecules is a mechanism for recurrence after haploidentical HSCT. However, previous studies involved patients with myeloid leukemia; no studies to date have examined HLA loss at relapse among patients with lymphoid leukemia or explored the incidence and risk factors associated with the antithymocyte globulin (ATG) transplant system. We conducted a multicenter retrospective case series study that aimed to provide initial data regarding the incidence of and characteristics associated with HLA loss at relapse among patients with lymphoid and myeloid leukemia after receipt of HSCT based on low-dose ATG T-cell–replete conditioning.

## Methods

This retrospective case series study was reviewed and approved by the ethics committee of the First Affiliated Hospital of Zhejiang University School of Medicine. Written informed consent was obtained from all patients or their parents before data collection. The study was conducted in accordance with the Declaration of Helsinki^14^ and the reporting guideline for case series.

### Patients

**Figure 1.**
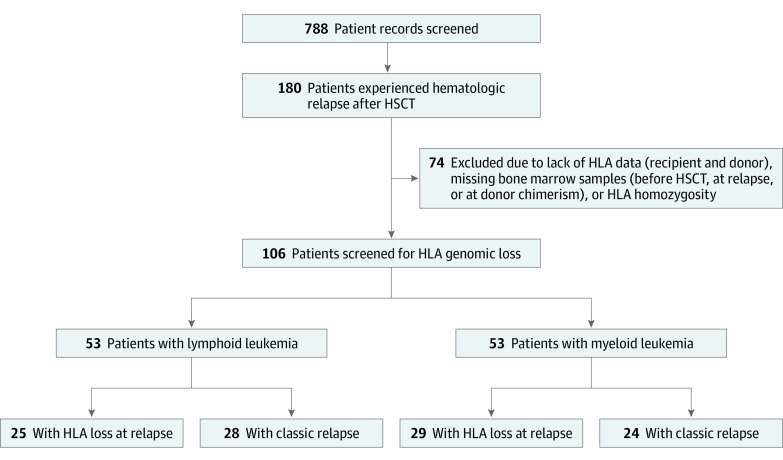
Study Flow Diagram Initial records search was performed for patients who had (1) hematologic cancer, (2) age between 8 and 70 years, (3) a low-dose antithymocyte globulin–based haploidentical hematopoietic stem cell transplant (HSCT), and (4) a transplant date between May 2012 and May 2021. A total of 788 records were identified and screened for patients with (1) minimal residual disease–negative during complete remission after haploidentical HSCT, (2) engraftment with 100% donor chimerism after haploidentical HSCT, (3) bone marrow hematologic relapse of minimal residual disease–positive, and (4) donor chimerism less than 97%. HLA indicates human leukocyte antigen.

The study included consecutive patients who received ATG T-cell–replete haploidentical HSCT at the Zhejiang Cooperative Group for Blood and Marrow Transplantation (including First Affiliated Hospital of Zhejiang University School of Medicine, Sir Run Run Shaw Hospital of Zhejiang University School of Medicine, Zhejiang Children Hospital, and People’s Hospital of Ningbo) between May 1, 2012, and May 31, 2021. An initial records search was performed to identify patients who had (1) hematologic cancer, (2) age between 8 and 70 years, (3) a low-dose ATG–based haploidentical HSCT, and (4) a transplant date between May 2012 and May 2021 (Figure 1). A total of 788 records were identified and screened. Patients included in the final analysis fully met the following criteria: (1) receipt of haploidentical HSCT for hematologic cancer, (2) achievement of minimal residual disease (MRD)–negative complete remission and successful engraftment with 100% donor chimerism after haploidentical HSCT, (3) decrease in donor chimerism (<97%) and MRD in bone marrow or hematologic relapse confirmed by bone marrow, (4) available bone marrow samples before transplant and at relapse as well as results of HLA typing for both patient and donor. Patients had received GVHD prophylaxis with cyclosporine, short course methotrexate, and mycophenolate mofetil.

### Covariates

The covariates were age, sex, disease type, pretransplant factors (underweight status, risk stratification, and previous treatments), transplant-related factors (donor age, sex, blood type compatibility, conditioning regimens, and ATG type), and posttransplant factors (GVHD and MRD positivity). Underweight status was defined as body mass index (BMI; calculated as weight in kilograms divided by height in meters squared) less than 18.5 and, among children and adolescents, fulfillment of underweight criteria from the screening standard for malnutrition of Chinese school-aged children and adolescents.^15^ Chronic GVHD was defined and graded according to criteria from the National Institutes of Health.^16^ Acute GVHD was defined and graded based on the Mount Sinai Acute GVHD International Consortium consensus.^17^ A posttransplant MRD-positive result was defined as the appearance of MRD positivity in the presence of fewer than 5% of cancer cells based on molecular and/or flow cytometry.

### Outcomes

The primary outcome in this study was HLA genome loss at the time of hematologic cancer relapse. Hematologic cancer relapse was defined as the presence of 5% or more cancer cells. Relapse was considered informative in patients who had at least 1 bone marrow sample exhibiting complete donor chimerism within 180 days before relapse.

The secondary outcome was postrelapse overall survival (OS), which was defined as the time from posttransplant relapse confirmed by bone marrow results until death associated with any cause. The date for the last follow-up censoring was July 1, 2021.

### HLA Typing and Genomic Loss Detection

The HLA typing of peripheral blood lymphocytes among patients and donors was conducted by the Blood Center of Zhejiang Province. Six loci (*HLA-A*, *HLA-B*, *HLA-C*, *HLA-DRB1*, *HLA-DQB1*, and *HLA-DPB1*) were used for the evaluation.

Patient-specific HLA genomic loss was defined as the loss of expression of patient-specific HLA loci in leukemic cells harvested in bone marrow. An HLA-KMR kit (GenDx) and an HLA loss detection kit (Shanghai Tissuebank Precision Medicine) were used for detecting HLA loss based on quantitative polymerase chain reaction testing.^18^ Patient-specific HLA and non-HLA markers were targeted. A total of 62 *HLA-A*, *HLA-B*, *HLA-C*, *HLA-DQB1*, *HLA-DRB1*, and *HLA-DPB1* alleles were evaluated. The diagnosis of HLA loss was confirmed when patient-specific HLA markers were negative (<3%), and non-HLA markers were positive (>3%). Among 21 patients without ad hoc–covered HLA loci and HLA homozygosity on detectable loci, next-generation sequencing was used for loss detection. Those without patient-specific HLA genomic loss were regarded as having classic relapse.

### Statistical Analysis

Univariate comparisons were conducted using a χ^2^, Fisher exact, or *t* test, as appropriate. Variables with *P* < .10 in univariate analysis and variables that might be clinically meaningful were entered into the logistic regression model (eTable 1 in the Supplement). The receiver operating characteristic curve was used to assess the diagnostic ability of HLA loss, with HLA loss at relapse vs classic relapse (reference variable) used as binary classifications. The Kaplan-Meier method was used to estimate the postrelapse OS of patients with either classic relapse (classic group) or HLA loss at relapse (HLA loss group). A log rank test was applied to evaluate differences between Kaplan-Meier curves. Univariate and multivariate Cox proportional hazard models were constructed to examine the hazard ratio (HR) for the different factors associated with death after relapse. Variables with *P* < .10 in the univariate Cox analysis and variables that might have been clinically meaningful were entered in the multivariate Cox proportional hazards model.

Data were analyzed using SPSS software, version 22.0.01 (IBM SPSS Statistics), and R software, version 3.4.3 (R Foundation for Statistical Computing). Two-sided *P* < .05 was considered statistically significant.

## Results

### Patient Characteristics

Between May 2012 and May 2021, 788 patients received haploidentical HSCT for hematologic cancer; of those, 180 patients experienced posttransplant relapse. A total of 106 evaluable patients (median age, 30.9 years [range, 8.3-64.6 years]; 54 female [50.9%] and 52 male [49.1%]) with available bone marrow samples and detailed clinical history were included in the final analysis (Table 1; Figure 1). The median follow-up after haploidentical HSCT was 18.0 months (range, 2.6-109.7 months). Of 106 patients, 104 experienced hematologic cancer relapse, and 2 experienced MRD recurrence. Patients were categorized in the classic or HLA loss group according to detection of the patient-specific HLA genome in leukemia cells. Among all patients, HLA loss was detected in 54 individuals (50.9%), and the occurrence of HLA loss was not associated with disease type (29 of 53 patients [54.7%] with myeloid leukemia vs 25 of 53 patients [47.2%] with lymphoid leukemia; *P* = .44).

**Table 1.** Patient, Donor, and Transplant Characteristics

Characteristic	No./total No. (%)					
	Myeloid cohort (n = 53)			Lymphoid cohort (n = 53)		
	HLA loss group	Classic group	*P* value	HLA loss group	Classic group	*P* value
**Patient**						
Total	29/53 (54.7)	24/53 (45.3)	NA	25/53 (47.2)	28/53 (52.8)	NA
Sex						
Male	16/24 (66.7)	8/24 (33.3)	.11	9/28 (32.1)	19/28 (67.9)	.02
Female	13/29 (44.8)	16/29 (55.2)		16/25 (64.0)	9/25 (36.0)	
Age, median (range), y	38.4 (8.3-60.2)	39.0 (20.3-58.5)	.89	27.0 (14.2-56.4)	26.1 (13.2-64.6)	.82
Diagnosis						
AML	20/39 (51.3)	19/39 (48.7)	.71	NA	NA	.69
MDS/CML	7/11 (63.6)	4/11 (36.4)		NA	NA	
CML	2/3 (66.7)	1/3 (33.3)		NA	NA	
B-ALL/LBL	NA	NA		21/46 (45.7)	25/46 (54.3)	
T-ALL/LBL	NA	NA		4/7 (57.1)	3/7 (42.9)	
WBC count at diagnosis (× 109/L)						
>50	5/9 (55.6)	4/9 (44.4)	>.99	8/18 (44.4)	10/18 (55.6)	.78
≤50	24/44 (54.5)	20/44 (45.5)		17/35 (48.6)	18/35 (51.4)	
Ratio of BM blasts at diagnosis, median (range)	0.61 (0.30-0.93)	0.61 (0.02-0.95)	.80	0.80 (0.30-0.95)	0.88 (0-0.96)	.03
AML gene variants						
* FLT3-ITD*	5/12 (41.7)	7/12 (58.3)	.20	NA	NA	NA
* WT1*	13/23 (56.5)	10/23 (43.5)	.82	NA	NA	NA
B-ALL/LBL						
Ph^−^	NA	NA	NA	14/30 (46.7)	16/30 (53.3)	>.99
Ph^+^	NA	NA		7/15 (46.7)	8/15 (53.3)	
B-ALL/LBL gene variant						
* IKZF1*	NA	NA	NA	4/9 (44.4)	5/9 (55.6)	>.99
* PAX5*	NA	NA		2/4 (50.0)	2/4 (50.0)	
Lines of induction chemotherapy, median (range)	2 (0-5)	1 (0-4)	.21	1 (1-5)	1 (0-4)	.28
Lines of chemotherapy before HSCT, median (range)	2 (0-5)	3 (1-7)	.42	3 (1-12)	3 (1-6)	.50
HMAs before HSCT	12/22 (54.5)	10/22 (45.5)	.99	NA	NA	NA
EMD before HSCT	0	1/1 (100)	NA	8/12 (66.7)	4/12 (33.3)	.14
Adoptive therapy before HSCT						
CAR T-cell therapy	1/1 (100)	0	NA	4/10 (40.0)	6/10 (60.0)	.74
Matched HSCT	0	1/1 (100)	NA	NA	NA	NA
Time from diagnosis to HSCT, median (range), d	244 (92-2380)	236 (62-2577)	.92	209 (92-1853)	245 (141-334)	.80
Disease status at HSCT						
CR1	12/24 (50.0)	12/24 (50.0)	.87	17/32 (53.1)	15/32 (46.9)	.49
≥CR2	5/9 (55.6)	4/9 (44.4)		6/14 (42.9)	8/14 (57.1)	
AD	12/20 (60.0)	8/20 (40.0)		2/7 (28.6)	5/7 (71.4)	
MRD-negative CR before HSCT	10/14 (71.4)	4/14 (28.6)	.14	18/32 (56.3)	14/32 (43.8)	.10
R-DRI risk category						
Low	1/1 (100)	0	.007	0	0	.66
Intermediate	12/28 (42.9)	16/28 (57.1)		17/34 (50.0)	17/34 (50.0)	
High	16/21 (76.2)	5/21 (23.8)		6/12 (50.0)	6/12 (50.0)	
Very high	0	3/3 (100)		2/7 (28.6)	5/7 (71.4)	
**Donor**						
Sex						
Male	19/37 (51.4)	18/37 (48.6)	.45	16/38 (42.1)	22/38 (57.9)	.24
Female	10/16 (62.5)	6/16 (37.5)		9/15 (60.0)	6/15 (40.0)	
Age, median (range), y	30.2 (17.3-53.7)	29.3 (16.2-58.5)	.69	43.4 (15.5-54.0)	38.6 (17.5-57.8)	.76
Donor to recipient						
Female to female	6/10 (60.0)	4/10 (40.0)	.43	7/7 (100)	0	.01
Female to male	4/6 (66.7)	2/6 (33.3)		2/8 (25.0)	6/8 (75.0)	
Male to male	12/19 (63.2)	7/19 (36.8)		7/20 (35.0)	13/20 (65.0)	
Male to female	7/18 (38.9)	11/18 (61.1)		9/18 (50.0)	9/18 (50.0)	
Relationship to recipient						
Parent	9/17 (52.9)	8/17 (47.1)	.68	14/28 (50.0)	14/28 (50.0)	.97
Child	14/24 (58.3)	10/24 (41.7)		5/12 (41.7)	7/12 (58.3)	
Sibling	3/8 (37.5)	5/8 (62.5)		5/11 (45.5)	6/11 (54.5)	
Other relative	3/4 (75.0)	1/4 (25.0)		1/2 (50.0)	1/2 (50.0)	
Donor-recipient crossmatching						
Major crossmatch incompatible	10/13 (76.9)	3/13 (23.1)	.07	3/8 (37.5)	5/8 (62.5)	.32
Minor crossmatch incompatible	3/7 (42.9)	4/7 (57.1)		4/8 (50.0)	4/8 (50.0)	
Major and minor crossmatch incompatible	3/3 (100)	0		3/3 (100)	0	
Crossmatch compatible	13/30 (43.3)	17/30 (56.7)		15/34 (44.1)	19/34 (55.9)	
HLA mismatched loci, median (range)	5 (4-5)	5 (3-5)	.60	5 (3-5)	5 (1-5)	.78
Pretransplant BMI						
Underweight	2/10 (20.0)	8/10 (80.0)	.01	9/13 (69.2)	4/13 (30.8)	.07
Not underweight	27/41 (65.9)	14/41 (34.1)		16/40 (40.0)	24/40 (60.0)	
Conditioning regimen						
MAC	26/49 (53.1)	23/49 (46.9)	.62	25/50 (50.0)	25/50 (50.0)	.24
RIC	3/4 (75.0)	1/4 (25.0)		0	3/3 (100)	
ATG type						
Genzyme (6 mg/kg/d)	17/34 (50.0)	17/34 (50.0)	.36	10/27 (37.0)	17/27 (63.0)	.13
Fresenius (10 mg/kg/d)	12/19 (63.2)	7/19 (36.8)		15/26 (57.7)	11/26 (42.3)	
MNC cells, median (range), ×10^8^/kg	12.5 (7.0-44.2)	13.9 (6.3-45.8)	.79	14.0 (5.3-32.0)	11.8 (3.5-23.3)	.10
CD34^+^ cells, median (range), ×10^6^/kg	5.3 (2.1-14.3)	6.1 (2.2-19.7)	.42	5.7 (1.5-17.9)	5.1 (1.5-15.4)	.52
Engraftment time, median (range), d						
Neutrophil	11 (8-19)	12 (10-19)	.19	12 (9-19)	13 (9-21)	.44
Platelet	12 (10-30)	14 (8-32)	.29	15 (10-44)	14 (8-36)	.94

Abbreviations: AD, active disease; ALL, acute lymphoblastic leukemia; AML, acute myeloid leukemia; ATG, antithymocyte globulin; B-ALL, B-cell acute lymphoblastic leukemia; BM, bone marrow; BMI, body mass index (calculated as weight in kilograms divided by height in meters squared); CAR, chimeric antigen receptor; CML, chronic myelocytic leukemia; CR, complete remission; EMD, extramedullary disease; HLA, human leukocyte antigen; HMA, hypomethylating agent; HSCT, hematopoietic stem cell transplant; LBL, lymphoblastic lymphoma; MAC, myeloablative conditioning; MDS, myelodysplastic syndrome; MNC, mononuclear cell; MRD, minimal residual disease; NA, not applicable; RIC, reduced intensity conditioning; R-DRI, Refined Disease Risk Index; T-ALL, T-cell acute lymphoblastic leukemia; WBC, white blood cell.

### HLA Loss at Relapse in the Myeloid Group

**Figure 2.**
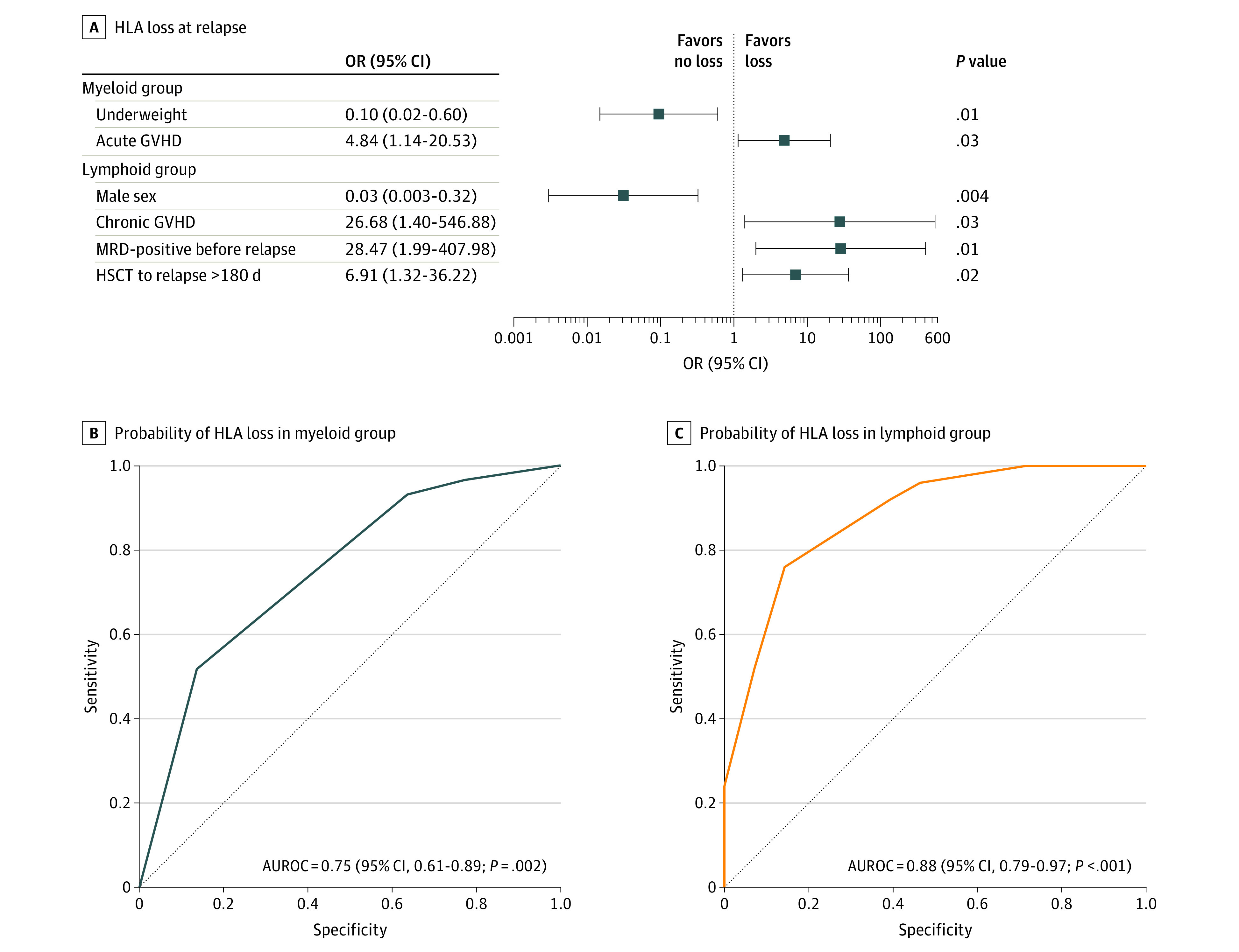
Independent Risk Factors Associated With Human Leukocyte Antigen Loss at Relapse AUROC indicates area under the receiver operating characteristic curve; GVHD, graft-vs-host disease; HLA, human leukocyte antigen; HSCT, hematopoietic stem cell transplant; and MRD, minimal residual disease.

A total of 53 patients with myeloid leukemia (51 with hematologic cancer relapse and 2 with donor chimerism and MRD positivity who died) were included in the analysis. The median age was 38.4 years (range, 8.3-60.2 years); 29 patients (54.7%) were female, and 24 (45.3%) were male. Patients in the HLA loss group vs the classic group were more likely to have a longer period between HSCT and relapse (median, 321 days [range, 55-1574 days] vs 223 days [range, 68-546 days]; *P* = .03) and to have GVHD (acute GVHD: 16 of 22 patients [72.7%] vs 6 of 22 patients [27.3%]; *P* = .03; chronic GVHD: 15 of 20 patients [75.0%] vs 5 of 20 patients [25.0%]; *P* = .02) (Table 2). Among those with myeloid leukemia, underweight status (odds ratio [OR], 0.10; 95% CI, 0.02-0.60; *P* = .01) and de novo acute GVHD (OR, 4.84; 95% CI, 1.14-20.53; *P* = .03) were associated with HLA loss at relapse (Figure 2A). The formula for HLA loss at relapse in the myeloid group was as follows: 0.157 subtracted from 2.354 multiplied by underweight status (value of 0 or 1, with 0 indicating no and 1 indicating yes) subtracted from 1.578 multiplied by de novo acute GVHD status (value of 0 or 1, with 0 indicating no and 1 indicating yes). The sensitivity and specificity of the HLA loss risk model were assessed using receiver operating characteristic analysis. The area under the curve was 0.75 (95% CI, 0.61-0.89; *P* = .002), indicating a diagnostic accuracy of 75.0% (Figure 2B).

**Table 2.** Characteristics After Transplant

Characteristic	No./total No. (%)					
	Myeloid cohort (n = 53)			Lymphoid cohort (n = 53)		
	HLA loss group	Classic group	*P* value	HLA loss group	Classic group	*P* value
Total	29/53 (54.7)	24/53 (45.3)	NA	25/53 (47.2)	28/53 (52.8)	NA
De novo acute GVHD	16/22 (72.7)	6/22 (27.3)	.03	12/25 (48.0)	13/25 (52.0)	.91
Time from HSCT to acute GVHD occurrence, median (range), d	25 (9-206)	38 (7-136)	.54	44 (5-166)	28 (7-228)	.69
Chronic GVHD	15/20 (75.0)	5/20 (25.0)	.02	8/9 (88.9)	1/9 (11.1)	.009
Time from HSCT to chronic GVHD occurrence, median (range), d	189 (119-1120)	197 (56-429)	>.99	189 (126-801)	657 (657-657)	.44
DLI before relapse	16/25 (64.0)	9/25 (36.0)	.20	6/9 (66.7)	3/9 (33.3)	.28
Cumulative CD3^+^ cells by DLI, median (range), ×10^7^/kg	2.0 (0.1-5.8)	1.5 (0.3-6.6)	.61	1.9 (1.0-2.8)	1.1 (1.0-1.8)	.30
MRD-positivity before relapse	17/28 (60.7)	11/28 (39.3)	.35	7/10 (70.0)	3/10 (30.0)	.16
Time from HSCT to relapse, median (range), y	321 (55-1574)	223 (68-546)	.03	323 (98-2056)	151 (57-2544)	.01
BM blasts ratio at relapse, median (range)	0.16 (0.01-0.89)	0.28 (0.06-0.92)	.04	0.40 (0-0.90)	0.30 (0-0.90)	.99
EMD at relapse	3/5 (60.0)	2/5 (40.0)	>.99	11/16 (68.8)	5/16 (31.3)	.04

Abbreviations: BM, bone marrow; DLI, donor lymphocyte infusion; EMD, extramedullary disease; GVHD, graft-vs-host disease; HLA, human leukocyte antigen; HSCT, hematopoietic stem cell transplant; MRD, minimal residual disease; NA, not applicable.

### HLA Loss at Relapse in the Lymphoid Group

A total of 53 patients with relapse of lymphoid leukemia were included in the analysis. The median age was 27.0 years (range, 13.2-64.6 years); 25 patients (47.2%) were female, and 28 (52.8%) were male. Most patients (46 individuals [86.8%]) had B-cell acute lymphoblastic lymphoma; of those, 9 patients (19.6%) received chimeric antigen receptor T-cell therapy before transplant. Only 3 of 53 patients (5.7%) received reduced-intensity conditioning.

The HLA loss group vs the classic group had a greater number of female patients (16 of 25 individuals [64.0%] vs 9 of 25 individuals [36.0%]; *P* = .02), a lower number of bone marrow blasts at diagnosis (median, 0.80 blasts [range, 0.30-0.95 blasts] vs 0.88 blasts [range, 0-0.96 blasts]; *P* = .03), and a slightly higher number of patients with MRD-negative complete remission before HSCT (18 of 32 individuals [56.3%] vs 14 of 32 individuals [43.8%]; *P* = .10) (Table 1). Regarding posttransplant parameters, patients in the HLA loss and classic groups had a similar incidence (12 of 25 individuals [48.0%] vs 13 of 25 individuals [52.0%]; *P* = .91) and time to onset (median, 44 days [range, 5-166 days] vs 28 days [range, 7-228 days]; *P* = .69) of de novo acute GVHD; however, a greater number of patients in the HLA loss group vs the classic group had chronic GVHD after HSCT (8 of 9 individuals [88.9%] vs 1 of 9 individuals [11.1%]; *P* = .009) (Table 2). Patients in the HLA loss group experienced later onset of hematologic cancer relapse (median, 323 days [range, 98-2056 days] vs 151 days [range, 57-2544 days]; *P* = .01) compared with patients in the classic group. Notably, patients in the HLA loss group were more likely to have concurrent extramedullary disease at relapse (11 of 16 individuals [68.8%] vs 5 of 16 individuals [31.3%]; *P* = .04) than those in the classic group.

Variables with *P* < .10 that were clinically meaningful before relapse were included in a logistic regression analysis to identify factors associated with HLA loss. This analysis revealed that patients were more likely to lose unshared HLA at relapse if they had MRD-positive results before relapse (OR, 28.47; 95% CI, 1.99-407.98; *P* = .01), chronic GVHD (OR, 27.68; 95% CI, 1.40-546.88; *P* = .03), or more than 180 days between HSCT and relapse (OR, 6.91; 95% CI, 1.32-36.22; *P* = .02). However, male patients (OR, 0.03; 95% CI, 0.003-0.32; *P* = .04) were more likely to have HLA genome preservation at relapse (Figure 2A). We established a formula for postulating HLA loss at relapse: −0.595 subtracted from 3.506 multiplied by male status (value of 0 or 1, with 0 indicating no and 1 indicating yes) plus 3.349 multiplied by MRD positivity before relapse (value of 0 or 1, with 0 indicating no and 1 indicating yes) plus 3.321 multiplied by chronic GVHD status (value of 0 or 1, with 0 indicating no and 1 indicating yes) plus 1.933 multiplied by relapse occurring more than 180 days after HSCT (value of 0 or 1, with 0 indicating no and 1 indicating yes). The sensitivity and specificity of the HLA loss risk model were assessed using receiver operating characteristic analysis. This analysis revealed that male sex, MRD positivity before relapse, chronic GVHD, and relapse occurring more than 180 days after HSCT could distinguish between HLA loss at relapse and classic relapse among patients with lymphoid leukemia (area under the curve, 0.88; 95% CI, 0.79-0.97; *P* < .001), indicating a diagnostic accuracy of 88.0% (Figure 2C).

### Preemptive Donor Lymphocyte Infusion and Postrelapse Treatment

Among all patients included in the retrospective analysis, 24 individuals in the HLA loss group and 14 individuals in the classic group had MRD positivity before relapse. Of those, 14 of 24 patients (58.3%) in the HLA loss group and 10 of 14 patients (71.4%) in the classic group received preemptive donor lymphocyte infusion (DLI). The receipt of preemptive DLI did not postpone hematologic recurrence after diagnosed MRD positivity in the HLA loss group (preemptive DLI vs no preemptive DLI: median, 322 days [range, 204-1030 days] vs 340 days [range, 215 days to not available (NA)]; *P* > .99) (eFigure 1A in the Supplement) or the classic group (preemptive DLI vs no preemptive DLI: median, 229 days [range, 149 days to NA] vs 444 days [range, 121 days to NA]; *P* = .54) (eFigure 1B in the Supplement). Postrelapse treatments received by patients in the HLA loss and classic groups are shown in eFigure 2 in the Supplement.

**Figure 3.**
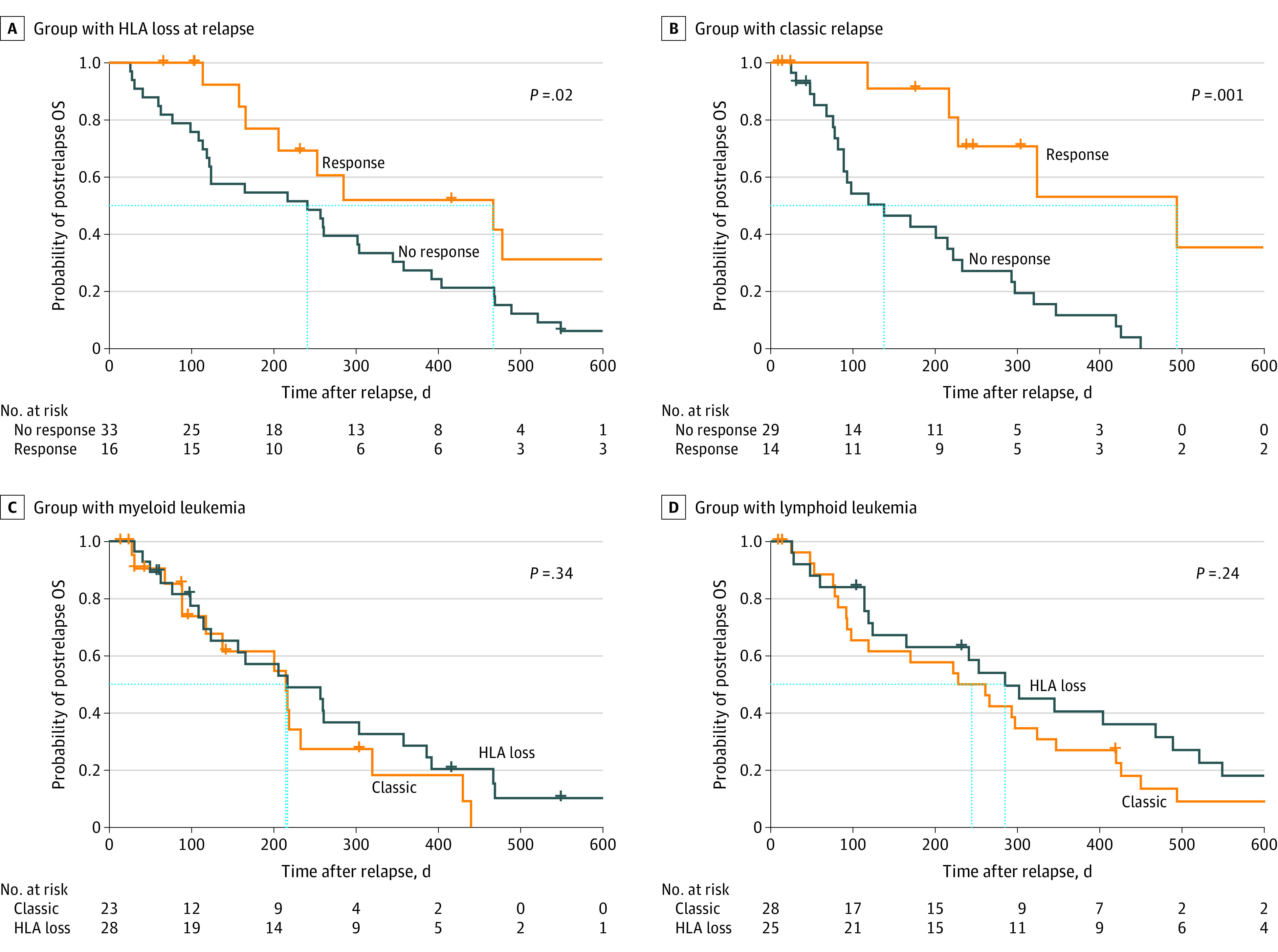
Postrelapse Overall Survival Plus signs indicate deaths occurring at specific time points. Boxes outlined by dashes indicate patients who were alive in each group at different time points. HLA indicates human leukocyte antigen; and OS, overall survival.

Achievement of response to salvage therapies was associated with survival benefits in the HLA loss group (response vs no response: median, 467 days [range, 206 days to NA] vs 241 days [range, 122-358 days]; *P* = .02) (Figure 3A) and the classic group (response vs no response: median, 494 days [range, 228 days to NA] vs 138 days [range, 89-293 days]; *P* = .001) (Figure 3B). Response rates were similar in the HLA loss group vs the classic group (17 of 31 patients [54.8%] vs 14 of 31 patients [45.2%]; *P* = .88). Seven patients (5 from the classic group and 2 from the HLA loss group) received a second HSCT from different donors, and only 2 patients in the HLA loss group were alive at the last follow-up (232 days and 386 days). Five patients in the classic group survived for 266 days, 324 days, 426 days, 494 days, and 650 days after relapse. Twelve patients with B-acute lymphoblastic leukemia received chimeric antigen receptor T-cell therapy, which did not produce a significant survival difference in the classic and HLA loss groups.

### Overall Survival in the Myeloid Group

The median postrelapse OS among those with myeloid leukemia was 217 days (range, 124-386 days) in the HLA loss group and 215 days (range, 118 days to NA) in the classic group (*P* = .34) (Figure 3C). The multivariate analysis revealed that only white blood cell count greater than 50 × 10^9^/L at diagnosis (HR, 7.50; 95% CI, 1.33-42.39; *P* = .02), female sex (HR, 0.12; 95% CI, 0.03-0.47; *P* = .003), receipt of myeloablative conditioning (HR, 0.03; 95% CI, 0.002-0.53; *P* = .02), and nonresponse to salvage therapies (HR, 0.09; 95% CI, 0.02-0.51; *P* = .006) were associated with lower postrelapse survival among patients with HLA loss vs no HLA loss at relapse (eTable 2B in the Supplement). The univariate and multivariate results regarding postrelapse OS in the classic group are available in eTable 2A and B in the Supplement.

### Overall Survival in the Lymphoid Group

The median postrelapse OS was 285 days (range, 165-521 days) in the HLA loss group and 244 days (range, 98-420 days) in the classic group (*P* = .24) (Figure 3D). The results of the univariate analysis of the HLA loss group revealed that male sex (HR, 2.58; 95% CI, 1.03-6.43; *P* = .04) and chronic GVHD (HR, 5.24; 95% CI, 1.68-16.27; *P* = .004) were associated with lower postrelapse OS (eTable 3A in the Supplement). The multivariate Cox regression analysis revealed that male sex (HR, 2.96; 95% CI, 1.13-7.76; *P* = .03) and the presence of chronic GVHD (HR, 6.27; 95% CI, 1.86-21.19; *P* = .003) were associated with death after HLA loss at relapse (eTable 3B in the Supplement). The univariate and multivariate results regarding postrelapse OS in the classic group are shown in eTable 3A and B in the Supplement.

## Discussion

This case series study found that HLA loss at relapse occurred frequently after ATG-based haploidentical HSCT. Our center and other major centers^19,20^ in China have previously found that haploidentical HSCT has advantages in reducing posttransplant relapse; these advantages might be associated with the mismatch of one-half of HLA molecules between patient and donor, in which donor T cells produce an intense allogeneic response and exert a substantial GVL effect.^21^ However, even among patients receiving haploidentical HSCT, the 2-year recurrence rate is 20% to 50%.^22,23,24^ The loss of patient-specific HLA is known to be one of the mechanisms associated with leukemic relapse after haploidentical HSCT.^25^ Because leukemic cells lose unshared HLA after haploidentical HSCT, the GVL effects are greatly reduced for T cells losing allogeneic targets. Among patients with HLA loss at relapse, frequent treatment with DLI may not produce GVL effects because of the lack of incompatible HLA targets in leukemic cells, but the recipient’s nonhematopoietic tissues (which have not undergone HLA loss) may continue to be attacked, ultimately leading to severe GVHD.^25^ Therefore, early detection of leukemic cells with patient-specific HLA loss, rather than undifferentiated application of HLA-restrictive interventions, may be beneficial for reducing unnecessary toxic effects from therapy.

Previous observational studies^12,13^ have reported that the rate of HLA loss at relapse after haploidentical HSCT among patients with myeloid leukemia is approximately 22.6% to 33.3%. In this study, we found disproportionate HLA loss at relapse among patients receiving stem cells grafted from haploidentical related donors with low-dose ATG. The reasons for the high incidence of HLA loss observed after haploidentical HSCT remain elusive and may be associated with the use of ATG prophylaxis as a T-cell–replete conditioning system that could conserve alloreactive T cells in the graft, which then produces immunological pressure and results in leukemic cells actively losing the genome of patient-specific HLA. Previous studies^12,13^ have reported an association between myeloid leukemia and HLA loss. However, we found that the risk factors associated with HLA loss at relapse were different among patients with myeloid leukemia; notably, this study was the first, to our knowledge, to identify elements of HLA loss at relapse among those with lymphoid leukemia.

Among patients with lymphoid leukemia, the possibility of HLA loss at relapse can be inferred based on the patient’s sex, the time from HSCT to relapse, the occurrence of MRD positivity, and the presence of chronic GVHD. The results of the present study suggested that male patients with lymphoid leukemia were less likely to have HLA loss at relapse, which was consistent with the findings of a recent study.^26^ Although sex is associated with many autoimmune disorders, the difference in immune regulation based on sex is unclear. One study^27^ found that sex can have implications for the selection and expansion of HLA-associated T cells. Among patients with the same autoimmune diseases, men have a more diverse T-cell receptor–variable β chain on CD8^+^ T cells than women, suggesting that male patients could develop more CD8^+^ T-cell clones in response to the same antigen stimulation.^27^ It can be assumed that, in the context of allogenic HSCT, donor cytotoxic T lymphocytes may yield greater variety in male recipients when encountering allogeneic antigens. In addition, because CD8^+^ T cells do not strictly rely on the HLA T-cell receptor complex for activation,^28^ CD8^+^ T cells from the donor have less pressure to winnow leukemic cells with patient-specific HLA than CD4^+^ T cells.

Our study observed a long period from transplant to relapse, and the appearance of molecular and cytogenetic relapse before morphological relapse suggested a dynamic balance between leukemic cells and cytotoxic T lymphocytes. Another study^29^ identified an interesting phenomenon in which chronic lymphocytic leukemia exhibited 2 distinct types of relapse. The study found that, among patients with early relapse, chronic lymphocytic leukemia cells were almost identical in genomic, transcriptional, and epigenetic features before and after transplant; among patients experiencing late relapse, leukemic cells exhibited heterogeneities in various aspects from pre-to posttransplant, which suggested an interaction between leukemic cells and immune stress.^29^ Hence, this study corroborated our finding that early relapse occurring after transplant was more likely to be a classic relapse, in which the relapsed clone may have been consistent with the pretransplant clone. In contrast, late relapse was more likely to lose unshared HLA that required prolonged immune stress and clonal evolution.

Unlike previous studies of myeloid conditions,^12,26^ we found that the pretransplant BMI of patients was associated with HLA loss; patients with lower BMI were less likely to lose unshared HLA at relapse. A previous study^30^ found that underweight patients had a higher rate of posttreatment relapse, which suggests underweight status could reflect the biological aggressiveness of leukemia. Given that physicians typically use actual body weight to calculate chemotherapy dose, the dose for underweight patients is generally lower than that of normal-weight or overweight patients. In addition, several studies^31,32^ have reported that nutritional status impacts the metabolism of chemotherapeutic agents. For example, 1 study^32^ found that nutritional status was associated with the level of cytochrome P450 enzymes, which are responsible for the metabolism of chemotherapeutic drugs. Thus, the relative increase in the aggressiveness of leukemic cells might be associated with relapse in underweight patients, mainly through rekindling of the original leukemic clones rather than a new leukemic subset produced by immune stress–mediated HLA loss.

Distinct characteristics in patients with lymphoid leukemia were associated with postrelapse OS in both the HLA loss and classic groups. Male patients and patients with chronic GVHD in the HLA loss group had lower postrelapse survival. Leukemia cells become invisible to donor T cells because of the loss of HLA, whereas HLA molecules sustain broad tissue expression, resulting in a separation of GVL and GVHD that presents as a shortened postrelapse OS because of GVHD toxic effects. Consistent with this process, the presence of GVHD was not associated with lower postrelapse OS in the classic group.

By aggregating data for myeloid and lymphoid leukemia (with data limited to analysis by specific disease), we found that preemptive DLI might not postpone hematologic cancer relapse in high-risk patients; these findings diverge from those of a previous study^33^ suggesting that preemptive DLI could improve disease-free survival. Because the present study was a case series, the results could not provide a comprehensive assessment of preemptive DLI treatment because of unavoidable bias. However, we could speculate that patients with HLA loss at relapse, who might eliminate unshared HLA molecules during the stage in which leukemia is undetectable (ie, the early MRD-positive period), may present no response to preemptive DLI. Notably, the use of postrelapse DLI appeared to be an acknowledged bias because of patients’ performance status, making the exploration of its efficiency in patients with HLA loss unsuitable.

### Limitations

This study has limitations. First, the study was of an observational and retrospective nature; therefore, its results should be interpreted with caution. Second, limited data substantially interfered with *P* values, which might produce substantial bias. Third, all detections were based on bone marrow samples because of technical limitations, resulting in an inability to diagnose HLA loss in patients with isolated extramedullary relapse. Fourth, we did not perform subgroup analyses of the benefits of various treatments for relapse because of the small number of patients and the heterogeneity of their diseases. In future studies, we plan to expand the number of participants to yield valid therapeutic results that may be used to guide postrelapse treatments or prophylaxis for HLA loss at relapse.

## Conclusions

This case series study used a model that accounted for both pretransplant variables and posttransplant events, which allowed assessment of patient-specific HLA loss at relapse. Overall, the study found that HLA loss at relapse occurred frequently after ATG-based haploidentical HSCT. However, because the study involved a limited number of patients, further validation of results in other centers is warranted. The results of this study may serve as an incentive for clinicians to consider optimal interventions among patients with any sign of relapse. Identification of risk factors associated with HLA loss at relapse would help to prompt screening of HLA loss, avoid potentially harmful infusions of donor T cells, and develop alternative therapeutic strategies.

## Notes

Supplement. **eTable 1.** Covariates Included in the Final Logistic Regression Models for Diagnosing HLA Loss at Relapse **eTable 2.** Univariate and Multivariate Analyses of prOS in Patients With Myeloid Cancer **eTable 3.** Univariate and Multivariate Analyses of prOS in Patients With Lymphoid Cancer **eFigure 1****.** High-Risk Patients With MRD Positivity **eFigure 2.** Flowchart of Postrelapse Therapies
