# Supplementary material for: Assessment of Patient-Specific Human Leukocyte Antigen Genomic Loss at Relapse After Antithymocyte Globulin–Based T-Cell–Replete Haploidentical Hematopoietic Stem Cell Transplant
Source: JAMA Netw Open. 2022 Apr 6;5(4):e226114. doi: 10.1001/jamanetworkopen.2022.6114 (PMC8987901; doi:10.1001/jamanetworkopen.2022.6114)
Supplement: Supplement. — eTable 1. Covariates Included in the Final Logistic Regression Models for Diagnosing HLA Loss at Relapse eTable 2. Univariate and Multivariate Analyses of prOS in Patients With Myeloid Cancer eTable 3. Univariate and Multivariate Analyses of prOS in Patients With Lymphoid Cancer eFigure 1. High-Risk Patients With MRD Positivity eFigure 2. Flowchart of Postrelapse Therapies [file jamanetwopen-e226114-s001.pdf]

# Supplementary Online Content

Wu H, Shi J, Luo Y, et al. Assessment of patient-specific human leukocyte antigen genomic loss at relapse after antithymocyte globulin–based T-cell–replete haploidentical hematopoietic stem cell transplant. *JAMA Netw Open*. 2022;5(4):e226114. doi:10.1001/jamanetworkopen.2022.6114

**eTable 1.** Covariates Included in the Final Logistic Regression Models for Diagnosing HLA Loss at Relapse

**eTable 2.** Univariate and Multivariate Analyses of prOS in Patients With Myeloid Cancer

**eTable 3.** Univariate and Multivariate Analyses of prOS in Patients With Lymphoid Cancer

**eFigure 1.** High-Risk Patients With MRD Positivity

**eFigure 2.** Flowchart of Postrelapse Therapies

This supplementary material has been provided by the authors to give readers additional information about their work.

**eTable 1**. Covariates Included in the Final Logistic Regression Models for Diagnosing HLA Loss at Relapse

| Variations | | | |
|---|---|---|---|
| | Myeloid malignancies group, No. (%) , n=53 (50) | | |
| | Loss<br>n=29 (54.7) | Classical<br>n=24 (45.3) | *P* value |
| Sex of patient | | | 0.11 |
| Male | 16/24 (66.7) | 8/24 (33.3) | |
| Female | 13/29 (44.8) | 16/29 (55.2) | |
| WBC at diagnosis (× $10^9$/L) | | | >0.99 |
| < 50 | 5/9 (55.6) | 4/9 (44.4) | |
| ≤ 50 | 24/44 (54.5) | 20/44 (55.6) | |
| Disease status at HSCT | | | 0.55 |
| CR | 17/33 (51.5) | 16/33 (48.5) | |
| AD | 12/20 (60.0) | 8/20 (40.0) | |
| R-DRI | | | 0.007 |
| Low | 1/1 (100.0) | 0/1 (0.0) | |
| Intermediate | 12/28 (42.9) | 16/28 (57.1) | |
| High | 16/21 (76.2) | 5/21 (23.8) | |
| Very high | 0/3 (0.0) | 3/3 (100.0) | |
| Pre-transplant BMI | | | 0.01 |
| Underweight | 2/10 (20.0) | 8/10 (80.0) | |
| No underweight | 27/41 (65.9) | 14/41 (34.1) | |
| De novo acute GVHD | | | 0.03 |
| No | 13/31 (41.9) | 18/31 (58.1) | |
| Yes | 16/22 (72.7) | 6/22 (27.3) | |
| Chronic GVHD | | | 0.02 |
| No | 14/33 (42.4) | 19/33 (57.6) | |
| Yes | 15/20 (75.0) | 5/20 (25.0) | |
| > 180 days from HSCT to relapse | | | 0.03 |
| No | 5/22 (22.7) | 17/22 (77.3) | |
| Yes | 20/31 (64.5) | 11/31 (35.5) | |
| | Lymphoid malignancies group, No. (%), n=53 (50) | | |
| | Loss<br>n=25 (47.2) | Classical<br>n=28 (52.8) | *P* value |
| Sex of patient | | | 0.02 |
| Male | 9/28 (32.1） | 19/28 (67.9) | |
| Female | 16/25 (64.0） | 9/25 (36.0) | |
| WBC at diagnosis (× $10^9$/L) | | | 0.78 |
| < 50 | 8/18 (44.4) | 10/18 (55.6) | |
| ≤ 50 | 17/35 (48.6) | 18/35 (51.4) | |

© 2022 Wu H et al. *JAMA Network Open*.

| | | | |
|---|---|---|---|
| BM blasts ratio at diagnosis above median (86%) | | | 0.07 |
| No | 17/29 (58.6) | 12/29 (41.4) | |
| Yes | 8/24 (33.3) | 16/24 (66.7) | |
| Disease status at HSCT | | | 0.43 |
| AD | 2/7 (28.6) | 5/7 (71.4) | |
| CR | 23/46 (50.0) | 23/46 (50.0) | |
| Donor to recipients | | | 0.01 |
| Female to female | 7/7 (100.0) | 0/7 (0.0) | |
| Female to male | 2/8 (25.0) | 6/8 (75.0) | |
| Male to male | 7/20 (35.0) | 13/20 (65.0) | |
| Male to female | 9/18 (50.0) | 9/18 (50.0) | |
| Pre-transplant BMI | | | 0.07 |
| Underweight | 9/13 (69.2) | 4/13 (30.8) | |
| No underweight | 16/40 (40.0) | 24/40 (60.0) | |
| MNC cells above median (12.4 × $10^8$/kg) | | | 0.13 |
| No | 10/27 (37.0) | 17/27 (63.0) | |
| Yes | 15/26 (57.7) | 11/26 (42.3) | |
| Chronic GVHD | | | 0.009 |
| No | 17/44 (38.6) | 27/44 (61.4) | |
| Yes | 8/9 (88.9) | 1/9 (11.1) | |
| MRD-positivity before relapse | | | 0.16 |
| No | 18/43 (41.9) | 25/43 (58.1) | |
| Yes | 7/10 (70.0) | 3/10 (30.0) | |
| > 180 days from HSCT to relapse | | | 0.003 |
| No | 5/22 (22.7) | 17/22 (77.3) | |
| Yes | 20/31 (64.5) | 11/31 (35.5) | |

Abbreviations: CI, confidence interval; HR, hazard ratio; prOS, postrelapse overall survival.

© 2022 Wu H et al. *JAMA Network Open*.

**eTable 2.** Univariate and Multivariate Analyses of prOS in Patients With Myeloid Cancer

A. Univariate analysis

| | Myeloid malignancies | | | | | |
|---|---|---|---|---|---|---|
| | Loss Group | | | Classical Group | | |
| Variations | HR | 95%CI | *P* value | HR | 95%CI | *P* value |
| Sex of patient | | | | | | |
| Female | Ref. | | 0.25 | Ref. | | 0.86 |
| Male | 0.611 | 0.262-1.426 | | 1.102 | 0.366-3.315 | |
| Age above median (44.5 years) | | | | | | |
| No | Ref. | | 0.18 | Ref. | | 0.03 |
| Yes | 0.544 | 0.223-1.326 | | 0.235 | 0.062-0.893 | |
| WBC at diagnosis (× $10^9$/L) | | | | | | |
| ≤ 50 | Ref. | | 0.05 | Ref. | | 0.48 |
| > 50 | 3.184 | 1.024-9.902 | | 1.643 | 0.420-6.431 | |
| FLT3-ITD variant | | | | | | |
| No | Ref. | | 0.25 | Ref. | | 0.13 |
| Yes | 1.816 | 0.655-5.039 | | 0.191 | 0.023-1.592 | |
| EMD before HSCT | | | | | | |
| No | - | - | - | Ref. | | 0.99 |
| Yes | - | - | - | 0.989 | 0.124-7.867 | |
| Disease status at HSCT | | | | | | |
| AD | Ref. | | 0.82 | Ref. | | 0.96 |
| CR | 1.106 | 0.461-2.651 | | 1.045 | 0.226-4.831 | |

© 2022 Wu H et al. *JAMA Network Open*.

| | | | | | | |
|---|---|---|---|---|---|---|
| MRD negative CR before HSCT | | | | | | |
| No | Ref. | | 0.21 | Ref. | | 0.81 |
| Yes | 1.748 | 0.737-4.149 | | 1.159 | 0.355-3.785 | |
| R-DRI | | | | | | |
| Low | Ref. | | 0.95 | - | - | - |
| Intermediate | 1.352 | 0.170-10.765 | | Ref. | | 0.02 |
| High | 1.405 | 0.178-11.085 | | 14.802 | 2.172-100.895 | |
| Very high | - | - | - | 2.744 | 0.304-24.753 | |
| Sex of donor | | | | | | |
| Female | Ref. | | 0.75 | Ref. | | 0.85 |
| Male | 1.166 | 0.455-2.985 | | 0.900 | 0.298-2.717 | |
| Donor to recipients | | | | | | |
| Female to female | Ref. | | 0.11 | Ref. | | 0.99 |
| Female to male | 2.183 | 0.411-11.601 | | 1.320 | 0.207-8.402 | |
| Male to male | 1.088 | 0.293-4.036 | | 1.011 | 0.216-4.737 | |
| Male to female | 4.057 | 0.940-17.518 | | 0.991 | 0.231-4.247 | |
| Pre-transplant BMI | | | | | | |
| Underweight | Ref. | | 0.78 | Ref. | | 0.23 |
| No underweight | 0.811 | 0.183-3.588 | | 0.437 | 0.114-1.672 | |
| Conditioning regimen | | | | | | |
| MAC | Ref. | | 0.10 | Ref. | | 0.57 |
| RIC | 0.181 | 0.024-1.357 | | 1.853 | 0.226-15.226 | |
| ATG type | | | | | | |
| ATG-G | Ref. | | 0.41 | Ref. | | 0.82 |
| ATG-F | 0.682 | 0.275-1.691 | | 1.133 | 0.385-3.334 | |

© 2022 Wu H et al. *JAMA Network Open*.

| | | | | | | |
|---|---|---|---|---|---|---|
| De novo acute GVHD | | | | | | |
| No | Ref. | | 0.79 | Ref. | | 0.82 |
| Yes | 1.124 | 0.479-2.639 | | 1.170 | 0.311-4.410 | |
| Chronic GVHD | | | | | | |
| No | Ref. | | 0.84 | Ref. | | 0.04 |
| Yes | 0.916 | 0.393-2.135 | | 4.710 | 1.118-19.846 | |
| DLI before relapse | | | | | | |
| No | Ref. | | 0.80 | Ref. | | 0.93 |
| Yes | 0.897 | 0.380-2.116 | | 0.951 | 0.304-2.975 | |
| HSCT to relapse > 180 days | | | | | | |
| No | Ref. | | 0.71 | Ref. | | 0.49 |
| Yes | 1.208 | 0.442-3.304 | | 0.690 | 0.240-1.987 | |
| BM blast > 20% at relapse | | | | | | |
| No | Ref. | | 0.16 | Ref. | | 0.61 |
| Yes | 1.884 | 0.787-4.510 | | 1.486 | 0.319-6.929 | |
| EMD at relapse | | | | | | |
| No | Ref. | | 0.21 | Ref. | | 0.64 |
| Yes | 0.391 | 0.091-1.704 | | 0.604 | 0.075-4.848 | |
| Response to salvage therapies | | | | | | |
| No | Ref. | | 0.14 | Ref. | | 0.21 |
| Yes | 0.464 | 0.169-1.276 | | 0.372 | 0.078-1.759 | |

Abbreviations: CI, confidence interval; HR, hazard ratio; prOS, postrelapse overall survival.

© 2022 Wu H et al. *JAMA Network Open*.

B. Multivariate analysis

| Myeloid malignancies | | | |
|---|---|---|---|
| Variations | HR | 95%CI | *P* value |
| Loss Group | | | |
| Sex of patient | | | |
| Female | Ref. | | |
| Male | 0.117 | 0.029-0.470 | 0.003 |
| WBC at diagnosis (× $10^9$/L) | | | |
| ≤ 50 | Ref. | | |
| > 50 | 7.501 | 1.328-42.386 | 0.02 |
| Conditioning regimen | | | |
| MAC | Ref. | | |
| RIC | 0.032 | 0.002-0.533 | 0.02 |
| Response to salvage therapies | | | |
| No | Ref. | | |
| Yes | 0.091 | 0.016-0.509 | 0.006 |
| Classical Group | | | |
| Age above median (44.5 years) | | | |
| No | Ref. | | 0.02 |
| Yes | 0.141 | 0.029-0.688 | |
| R-DRI | | | |
| Low | - | - | - |
| Intermediate | Ref. | | 0.04 |
| High | 40.380 | 3.668-444.59 | 0.003 |

© 2022 Wu H et al. *JAMA Network Open*.

| | | | |
|---|---|---|---|
| Very high | 1.298 | 0.140-12.056 | 0.82 |
| Chronic GVHD | | | |
| No | Ref. | | 0.46 |
| Yes | 3.338 | 0.141-79.297 | |

Abbreviations: CI, confidence interval; HR, hazard ratio; prOS, postrelapse overall survival.

© 2022 Wu H et al. *JAMA Network Open*.

**eTable 3.** Univariate and Multivariate Analyses of prOS in Patients With Lymphoid Cancer

A. Univariate analysis

| | Lymphoid malignancies | | | | | |
|---|---|---|---|---|---|---|
| | Loss Group | | | Classical Group | | |
| Variations | HR | 95%CI | *P* value | HR | 95%CI | *P* value |
| Sex of patient | | | | | | |
| Female | Ref. | | 0.04 | Ref. | | 0.41 |
| Male | 2.575 | 1.030-6.437 | | 0.692 | 0.290-1.648 | |
| Age above median (27.0 years) | | | | | | |
| No | Ref. | | 0.90 | Ref. | | 0.64 |
| Yes | 1.057 | 0.444-2.515 | | 1.221 | 0.534-2.789 | |
| WBC at diagnosis ( $\times 10^9$/L) | | | | | | |
| ≤ 50 | Ref. | | 0.95 | Ref. | | 0.53 |
| > 50 | 1.027 | 0.421-2.504 | | 0.749 | 0.303-1.847 | |
| CAR T-cell therapy before HSCT | | | | | | |
| No | Ref. | | 0.09 | Ref. | | 0.18 |
| Yes | 2.783 | 0.850-9.113 | | 2.101 | 0.704-6.267 | |
| EMD before HSCT | | | | | | |
| No | Ref. | | 0.87 | Ref. | | 0.29 |
| Yes | 0.925 | 0.357-2.396 | | 0.506 | 0.142-1.807 | |
| Disease status before HSCT | | | | | | |
| AD | Ref. | | 0.34 | Ref. | | 0.48 |
| CR | 0.477 | 0.105-2.161 | | 1.488 | 0.500-4.429 | |

© 2022 Wu H et al. *JAMA Network Open*.

| | | | | | | |
|---|---|---|---|---|---|---|
| MRD negative CR before HSCT | | | | | | |
| No | Ref. | | 0.93 | Ref. | | 0.76 |
| Yes | 0.958 | 0.370-2.481 | | 1.135 | 0.496-2.602 | |
| R-DRI | | | | | | |
| Low | - | - | - | - | - | - |
| Intermediate | Ref. | | 0.54 | Ref. | | 0.21 |
| High | 1.342 | 0.480-3.752 | | 2.565 | 0.802-8.206 | |
| Very high | 2.232 | 0.483-10.309 | | 0.790 | 0.256-2.438 | |
| Sex of donor | | | | | | |
| Female | Ref. | | 0.98 | Ref. | | 0.87 |
| Male | 1.012 | 0.389-2.630 | | 0.924 | 0.355-2.404 | |
| Donor to recipients | | | | | | |
| Female to female | Ref. | | 0.04 | - | - | - |
| Female to male | 12.960 | 1.908-88.029 | | Ref. | | 0.62 |
| Male to male | 2.251 | 0.642-7.887 | | 0.737 | 0.249-2.184 | |
| Male to female | 1.070 | 0.319-3.593 | | 1.200 | 0.411-3.503 | |
| Pre-transplant BMI | | | | | | |
| Underweight | Ref. | | 0.17 | Ref. | | 0.54 |
| No underweight | 0.532 | 0.217-1.305 | | 1.411 | 0.474-4.202 | |
| Conditioning regimen | | | | | | |
| MAC | - | - | - | Ref. | | 0.50 |
| RIC | - | - | - | 1.524 | 0.444-5.223 | |
| ATG type | | | | | | |
| ATG-G | Ref. | | 0.16 | Ref. | | 0.26 |
| ATG-F | 0.518 | 0.207-1.296 | | 0.616 | 0.265-1.431 | |

© 2022 Wu H et al. *JAMA Network Open*.

| | | | | | | |
|---|---|---|---|---|---|---|
| De novo acute GVHD | | | | | | |
| No | Ref. | | | Ref. | | |
| Yes | 0.587 | 0.242-1.422 | 0.24 | 0.497 | 0.215-1.149 | 0.10 |
| Chronic GVHD | | | | | | |
| No | Ref. | | | Ref. | | |
| Yes | 5.235 | 1.684-16.268 | 0.004 | 0.044 | 0.000-132.975 | 0.45 |
| DLI before relapse | | | | | | |
| No | Ref. | | | Ref. | | |
| Yes | 2.302 | 0.798-6.642 | 0.12 | 5.090 | 1.286-20.148 | 0.02 |
| HSCT to relapse > 180 days | | | | | | |
| No | Ref. | | | Ref. | | |
| Yes | 1.360 | 0.492-3.754 | 0.55 | 0.243 | 0.093-0.637 | 0.004 |
| EMD at relapse | | | | | | |
| No | Ref. | | | 1 | | |
| Yes | 1.149 | 0.484-2.725 | 0.75 | 0.452 | 0.133-1.534 | 0.20 |
| BM blast > 20% at relapse | | | | | | |
| No | Ref. | | | Ref. | | |
| Yes | 1.206 | 0.496-2.935 | 0.68 | 0.987 | 0.428-2.278 | 0.98 |
| Response to salvage therapies | | | | | | |
| No | Ref. | | | Ref. | | |
| Yes | 0.362 | 0.103-1.272 | 0.11 | 0.164 | 0.037-0.732 | 0.02 |

Abbreviations: CI, confidence interval; HR, hazard ratio; prOS, postrelapse overall survival.

© 2022 Wu H et al. *JAMA Network Open*.

B. Multivariate analysis

| Lymphoid malignancies | | | |
|---|---|---|---|
| Variations | HR | 95%CI | *P* value |
| Loss Group | | | |
| Sex of patient | | | |
| Female | Ref. | | 0.03 |
| Male | 2.964 | 1.132-7.758 | |
| Chronic GVHD | | | |
| No | Ref. | | 0.003 |
| Yes | 6.271 | 1.856-21.191 | |
| Classical Group | | | |
| Response to salvage therapies | | | |
| No | Ref. | | 0.02 |
| Yes | 0.164 | 0.037-0.732 | |

© 2022 Wu H et al. *JAMA Network Open*.

**eFigure 1.** High-Risk Patients With MRD Positivity

High-risk patients with MRD positivity might not prevent from relapse when using preemptive donor lymphocyte infusion (pDLI) in the (A) Loss Group and (B) Classical Group.

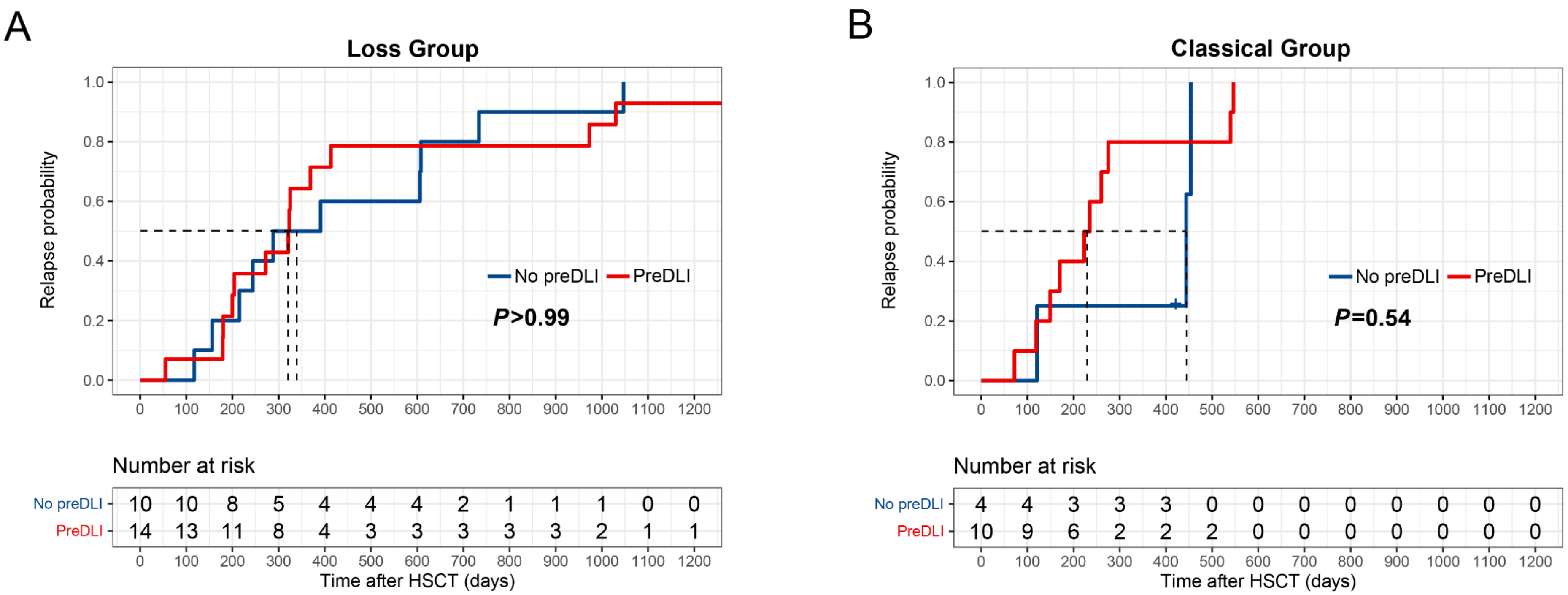


Abbreviation: HSCT, hematopoietic stem cell transplantation.

© 2022 Wu H et al. *JAMA Network Open*.

**eFigure 2.** Flowchart of Postrelapse Therapies

Post-relapse treatments for (A) myeloid disease and (B) lymphoid disease were presented

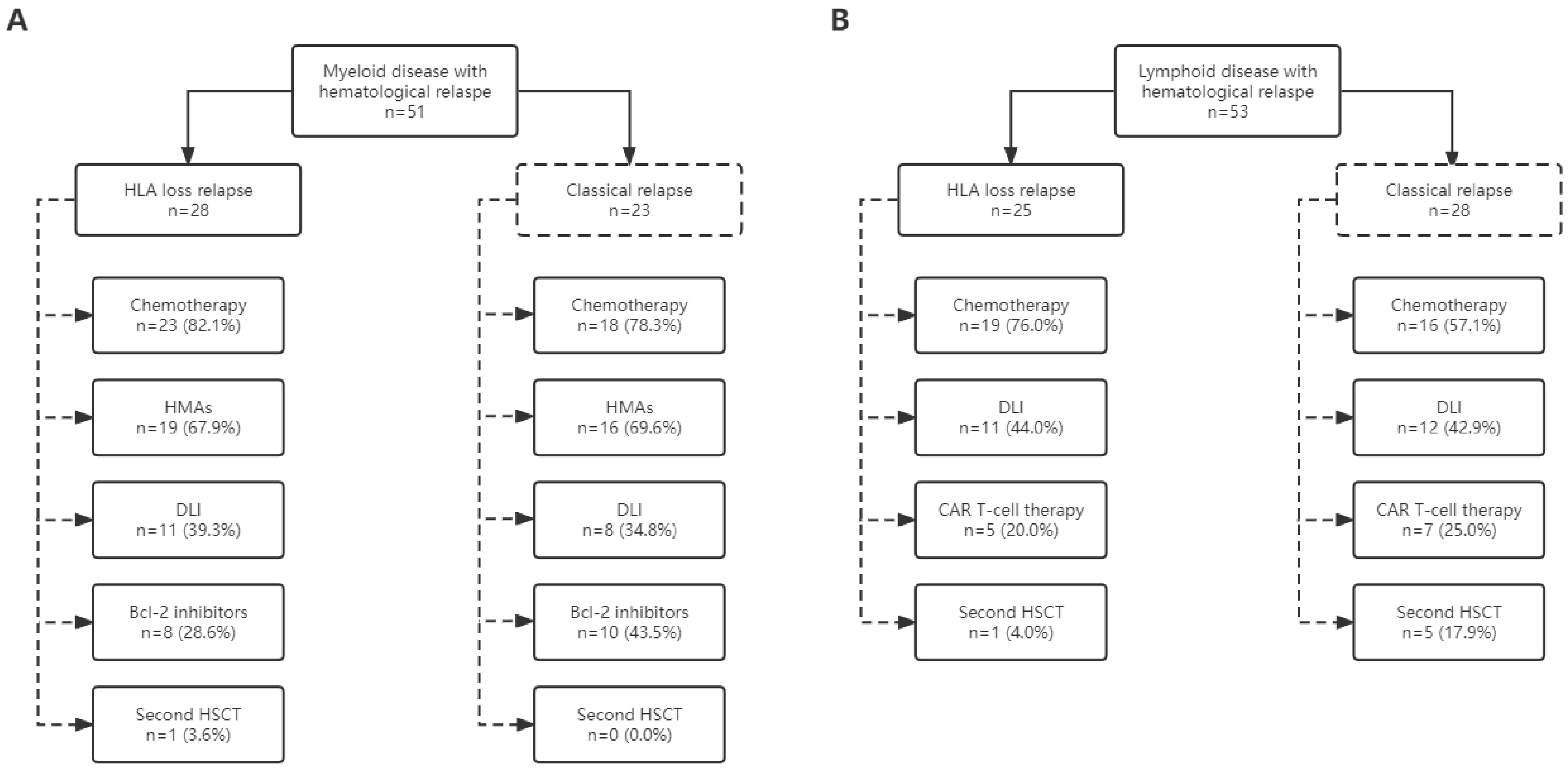


Abbreviations: DLI, donor lymphocyte infusion; HMA, hypomethylating agents; CAR, chimeric antigen receptor.

© 2022 Wu H et al. *JAMA Network Open*.
